# Attraction Basins as Gauges of Robustness against Boundary Conditions in Biological Complex Systems

**DOI:** 10.1371/journal.pone.0011793

**Published:** 2010-08-05

**Authors:** Jacques Demongeot, Eric Goles, Michel Morvan, Mathilde Noual, Sylvain Sené

**Affiliations:** 1 TIMC–IMAG, University Joseph Fourier of Grenoble, CNRS UMR 5525, La Tronche, France; 2 University Adolfo Ibañez, Peñalolen, Santiago, Chile; 3 ISCV, Complex Systems Institute of Valparaiso, Valparaiso, Chile; 4 Veolia Environment Research and Innovation, Rueil-Malmaison, France; 5 Santa Fe Institute, Santa Fe, New Mexico, United States of America; 6 LIP, University of Lyon, ÉNS-Lyon, CNRS UMR 5668, Lyon, France; 7 IBISC, University of Évry, CNRS FRE 3190, Évry, France; 8 IXXI, Rhône-Alpes Complex Systems Institute, Lyon, France; Center for Genomic Regulation, Spain

## Abstract

One fundamental concept in the context of biological systems on which researches have flourished in the past decade is that of the apparent robustness of these systems, i.e., their ability to resist to perturbations or constraints induced by external or boundary elements such as electromagnetic fields acting on neural networks, micro-RNAs acting on genetic networks and even hormone flows acting both on neural and genetic networks. Recent studies have shown the importance of addressing the question of the environmental robustness of biological networks such as neural and genetic networks. In some cases, external regulatory elements can be given a relevant formal representation by assimilating them to or modeling them by boundary conditions. This article presents a generic mathematical approach to understand the influence of boundary elements on the dynamics of regulation networks, considering their attraction basins as gauges of their robustness. The application of this method on a real genetic regulation network will point out a mathematical explanation of a biological phenomenon which has only been observed experimentally until now, namely the necessity of the presence of gibberellin for the flower of the plant *Arabidopsis thaliana* to develop normally.

## Introduction

Understanding certain phenomena emerging from the dynamical behaviour of complex dynamical systems, such as properties of auto-organisation and the ability to adapt to natural constraints and perturbations, are intimately related to the question of their structural robustness. This notion seems all the more pertinent in the field of biological complex networks that are modeled by discrete dynamical systems. We identify three kinds of robustnesses which we believe to be amongst the most relevant to study because of their usefulness in achieving a better understanding of regulation principles: environmental robustness (*i.e.*, the ability of a network to resist to external influences) [1]–[4], dynamical robustness (*i.e.*, the ability of a network to conserve the same asymptotic dynamics depending on underlying iteration modes) [5]–[8] and topological robustness (*i.e.*, the global dynamical stability of a network when it is submitted to structural perturbations and according to the existence of specific structural patterns, such as positive and negative circuits, which are recurrent in biological networks) [9]–[11].

The purpose of this paper is to focus on a kind of robustness that may be considered as an instance of the first kind of robustness mentioned above, namely robustness against perturbations induced by boundary elements that act on the system but are not modified by it. The motivation for studying boundary conditions of a network comes from the fact that boundary elements of a biological regulation network (for instance electric and magnetic fields in the context of neural networks, micro-RNAs and hormone flows in the context of genetic networks) may be seen as boundary elements acting on the intrinsic regulation of the network.

There is a classical view considering that the boundary between a cell and its environment is an anatomic boundary like the cytoplasmic membrane: in the case of a plant, it has been shown that flows of hormones, such as Auxin, propagate from cell to cell by crossing the cellular membrane, accelerating cell proliferation and improving the metabolic pathways that transform the nutrients necessary for the plant development [12]–[14]. In the approach presented in the sequel, the notion of boundary is related to the topology of the interaction graph associated to the regulation network. If, for instance, at a certain time of the biological dynamics, the co-expressed genes belong to a defined part of the chromatin, the boundary of the corresponding block of genes is the set of genes whose product of expression belong to the set of the regulators of the block [15], [16] maintaining “the correct spatial and temporal epigenetic code within the eukaryotic genome” [17]. Thus, we believe that studying the impact of stable topological boundaries in biological regulation systems modeling specific physiological functions is a relevant way to refine our understanding of real systems. The approach we present here is set at the frontier between discrete mathematics, theoretical computer science and biology. It is based on the idea that attraction basins of the dynamical behaviour of a network yield information on how the network operates and evolves. We show how an analysis of the influence of boundary elements on the asymptotic dynamical behaviour of a discrete dynamical system may profit from the observation of the variations of its attraction basins. To highlight the pertinence of our method, all its different steps are applied to an illustrative “toy regulation network” that models the genetic regulation of the floral morphogenesis of plant *Arabidopsis thaliana*. The choice of this particular network will yield a formal explanation of a phenomenon only observed experimentally until now [18], [19] (namely, the necessity of gibberellin to the normal development of the flower of *Arabidopsis thaliana*). In this network, the gibberellin will be considered as belonging to the functional (topological) boundary of the interaction graph (even if it acts also through the cell anatomic boundary). Our principal objective, however, is to present a multi-disciplinary method to analyse the robustness of an arbitrary biological complex network, and more generally of any kind of discrete dynamical system, against perturbations induced by its boundary and possibly, external elements.

The first section gives the main preliminary definitions that are used in this document. It specifically focuses on two notions: attractors/attraction basins of discrete dynamical systems, and centres/boundaries of networks. It also defines the model of regulation network on which this study is based. The following section describes our toy network, *i.e.*, the network we chose to serve as our case study. The different measures used to highlight relationships between boundary conditions and attraction basins are explained in the third section. These measures are used to draw some results on the effect that the boundary element (gibberellin) of our toy network has on its dynamics. The last section deals with stochastic state perturbations. An algorithm is proposed to study the robustness of a system against random state perturbations using attraction basins features. Again, this algorithm is applied to the floral morphogenesis genetic regulation network of plant *Arabidopsis thaliana*. Our paper ends with a discussion on the main perspectives of this work and some concluding remarks.

## Materials and Methods

### Preliminary Definitions

The objective of this section is to deliver some basic definitions from discrete dynamical systems theory and graphs theory before detailing the mathematical model used in our work to represent the dynamical evolution of genetic regulation networks.

### Dynamical System, Attractor and Attraction Basin

A discrete dynamical system 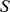 is a system composed by elements that interact with each other over time. More formally, a discrete dynamical system is defined by a triple 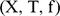, where:

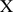
 is a discrete finite set, called the space of configurations.

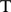
 equals 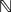 and is called the time space.

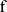
 is a map 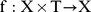 and satisfies 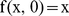 and 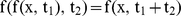.

**Figure 1:**
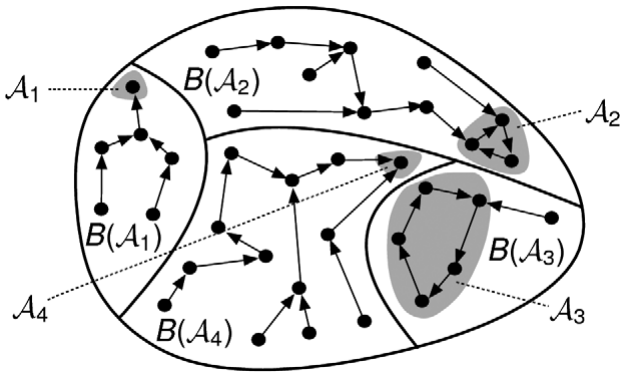
Iteration graph. Iteration graph representing the dynamics of an arbitrary discrete dynamical system having four attractors : two fixed points, 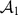 and 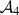, and two limit cycles, 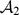 and 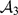. The attraction basins of these attractors are respectively 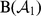, 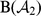, 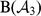 and 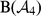.

In the following, we will only consider discrete dynamical systems where the map 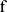, called the *flow* or the *global transition function* of the system, is a deterministic function. Let us consider a configuration 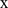 of 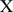 and apply a flow 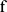 to it successively. Since the space of configurations is a finite set, whatever the deterministic flow 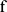 is, it is trivial that 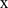 evolves in a finite time towards either a configuration which cannot evolve anymore, *i.e.*, a *fixed point*, or a sequence of configurations which repeat themselves indefinitely, *i.e.*, a *limit cycle*. Fixed points and limit cycles are called the *attractors* of the system [20], [21]. The number of configurations of an attractor is called the *period* of this attractor. Thus, a fixed point is an attractor of period 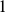 and a limit cycle containing 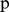 configurations is an attractor of period 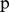. The set of configurations that evolve towards an attractor 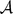 is called the *attraction basin* of 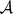 and is noted 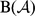. For any attractor 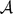, 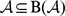. Let 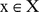 be an arbitrary configuration of a discrete dynamical system 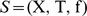. The sequence of configurations (including 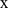) obtained by successive applications of 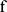 is called a *trajectory* of 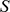. We can represent the trajectories of all the configurations 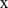 of 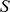 by an *iteration graph*. An illustration of the iteration graph of an arbitrary discrete dynamical system is pictured in Figure 1. In this figure, black dots represent configurations and arrows represent transitions between configurations resulting from the application of the global transition function of the system.

### Directed Graph, Centre and Boundary

In this article, we focus on *genetic regulation networks* which are particular discrete dynamical systems. These networks have been developed to model interactions dynamics occurring over time between genes. The structure of a regulation network 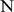 is generally represented by a directed graph 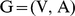, called *interaction graph*, where 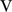 is the set of vertices (genes) and 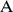 is the set of arcs (interactions between genes). Let us recall some useful definitions of graph theory in our context [22].

Let 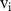 and 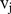 be two distinct vertices of a regulation network 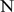 whose interaction graph is 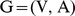. 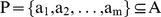 is a *path* from 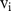 to 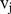 if the beginning of the arc 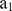 is the vertex 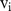, the end of the arc 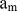 is the vertex 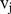 and the final vertex of each arc of 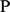 is the beginning vertex of the next arc of 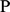. The *length* of a path equals the number of arcs that compose it.

The 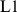-*distance* between two vertices 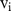 and 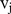, denoted by 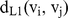 is the length of the shortest path from 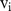 to 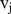. The *eccentricity* of a vertex 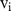 is the maximum 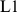-distance between 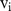 and any other vertex of the graph 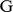. When a vertex 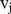 is not accessible from 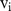, we have: 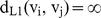. The minimum and non null eccentricity of the graph is called the graph *radius* and the maximum eccentricity of 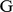 is called the *diameter* of the graph. The *centre* of a graph 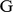 is the set of vertices whose eccentricity equals the graph radius. We will say that such vertices are *central*. We also define the *boundary* of a graph 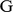 as the set of source vertices, that is, vertices with no arcs incoming from other vertices than themselves.

Let us add that the computation of the centre of an arbitrary graph corresponds to the computation of all the shortest paths for all the oriented couples of vertices. So, in the general case, it needs a time complexity of 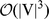 using the algorithm of Dijkstra [23] for each vertex. In the case of sparse graphs, *i.e.*, where 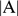 is significantly less than 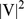, this time complexity can be reduced to 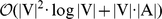 thanks to the algorithm of Johnson [24].

### Threshold Boolean Automata Networks as a Model of Regulation Networks Dynamics

In [25], McCulloch and Pitts introduced the model of threshold Boolean automata networks, also known as formal (or artificial) neural networks. Its purpose was to model the logical properties of the interactions between neurons from the point of view of discrete mathematics. A particular case of this model was then studied by Hopfield in [26], [27] in the context of physics. It was shown to present collective computational abilities which seemed to show a good correspondence with real neural networks. More precisely, Hopfield highlighted the notions of memory and learning. At the same time, in the field of discrete mathematics, researchers studied the asymptotic dynamical behaviour of threshold Boolean automata networks. They noticed some interesting properties, such as the importance of the iteration mode (this notion will be discussed later) and the nature of the attractors for specific networks [28], . On the other hand, in the context of genetic regulation networks modeling, two reference models, using different formalisms of two different levels of abstraction, were introduced a decade before: that of Kauffman at the end of the 1960's [30], and that of Thomas at the beginning of the 1970's [31]. Since then, many studies have been performed on the dynamical properties of both these models. To obtain more details on this subject, the reader can refer to [11], [32]–[34], assuming that this list is not exhaustive. According to us, threshold Boolean automata networks constitute a relevant model in this field of genetic regulation networks. Of course, our claim here is not to argue that this mathematical model allows a perfect representation of the biological reality (*e.g.* there is no consideration of spatial aspects) but that it allows to represent genes interactions at a certain level of abstraction which provides an interesting theoretical framework. Let us notice that this model was first used at the end of the 1990's in the context of genetic regulation [35] to model the floral morphogenesis of the plant *Arabidopsis thaliana*.

In this work, we have decided to focus on threshold Boolean automata networks whose evolution is governed by a deterministic updating rule. In this context, a network 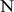 is a set of 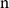 nodes which interact over time. Each node has two possible states, named activity states. If we call 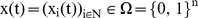 the current configuration of the network 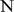 at time 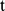, the states of the nodes of this configuration are defined by:
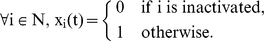
As mentioned earlier, the structure of a threshold Boolean automata network 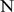 can be represented by a labelled directed graph called its *interaction graph*. In this graph, each arc 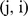 is labelled by an *interaction weight*, 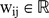. The sign of 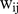 depends on the activating or inhibiting nature of the interaction that node 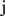 has on node 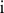. If 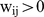 (resp. 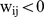), then node 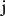 is said to be an *activator* (resp. a *repressor*) of node 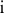. If 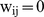, then node 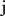 does not act on node 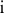 (and the arc 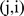 does not exist in the interaction graph of the network). Let us write 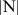 the number of nodes of the network 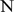 and 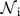 to refer to the neighbourhood of node 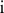, that is, the set of nodes which are activators or repressors of node 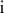. Then, 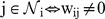 (which is also equivalent to the arc 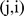 belonging to the network interaction graph). We define the *interaction matrix* 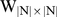 (also called the synaptic weights matrix in the context of neural networks) of the network. Its coefficient 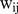 is the interaction weight that node 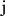 has on node 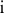. In the interaction graph of the network, each node is also labelled by a value called its *activation threshold*. It represents the necessary quantity of interaction potential a node needs to become activated. We define the 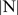-dimensional vector 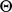 as the *threshold vector* in which the coefficient 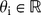 gives the activation threshold of node 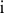.

Now, we can describe the temporal evolution of a threshold Boolean automata network. Informally, the new state of an arbitrary node 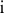 at time step 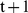 depends on the sum of the interaction weights coming from its activated neighbours at time step 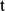. If this sum is greater than the activation threshold 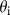, then the new state of node 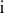 equals 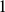. It equals 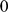 otherwise. Formally, the local transition function is defined by:
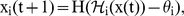
where 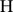 is the Heaviside step function (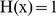 if 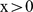 and 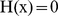 if 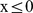) and 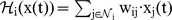 is the *interaction potential* of node 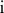.

A question that unavoidably rises when one studies the dynamical behaviour of a threshold Boolean automata network is that of the choice of an iteration mode, that is, the order according to which the local transition functions of the nodes are executed in order to update their states. Traditionally, studies on these kinds of networks have chosen either the *parallel iteration mode* (at each time step, the states of all nodes are updated simultaneously as suggested in the definition of local transition function given above) or a *sequential iteration mode* (at each time step, the state of one node is updated, which node that is depends on a predefined ordering of the nodes). These two particular iteration modes are also known respectively as the *totally synchronous* and the *asynchronous* iteration modes. A more general iteration mode which can be used is a *block-sequential iteration mode*: nodes are grouped into disjoint blocks; the states of nodes belonging to a same block are updated in parallel while the blocks themselves are updated sequentially. This kind of iteration mode is also called *partially synchronous* (or even *synchronous*) in other contexts. Parallel and sequential iteration modes are particular cases of block-sequential iteration modes. The number of block-sequential iteration modes in a network composed of 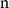 nodes equals the number of ordered partitions of a set of cardinal 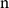. Thus, if we denote by 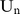 the number of block-sequential iteration modes for a network composed of 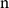 nodes, we have (see [36]):
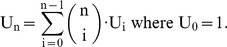
Understanding the precise impact of iteration modes on networks is, however, not the central objective of this work. For more information on this problematic, the reader can refer to [5], [6], [8], [28], [29].

### Model of the Floral Morphogenesis Genetic Regulation Network

As explained above, our aim is to propose a method to study the influence of boundary conditions on genetic regulation networks. To describe this method here we have chosen to apply it to the analysis of an illustrative network. All key notions, however, are described in a generic manner and every step of the analysis we present can be generalised in order to examine methodically how boundary elements act on any real genetic regulation network. The network we chose to serve as our “toy model” is that of the genetic regulation of the floral morphogenesis of the plant *Arabidopsis thaliana*. Working on this network will allow us, in particular, to explain formally a real biological phenomenon observed only experimentally until now: the influence of gibberellin on the development process of the flower of *Arabidopsis thaliana*. The influence of this hormone will be explained later by studying how its presence or absence acts on the asymptotic dynamical properties of the underlying network. Before that, in this section, we present the network. More precisely, we first present the original genetic regulation network of the floral morphogenesis of *Arabidopsis thaliana*, as it was introduced by Mendoza and Alvarez-Buylla [35] in 1998. To highlight some of its dynamical properties, we show it to be equivalent, in a way that we explicit later, to a simpler network that we call *reduced Mendoza & Alvarez-Buylla network*. Using this reduced network, we will introduce the notion of general iteration graph. This will allow us to explain our choice of a particular iteration mode used in the sequel. Then, we describe the variant, inspired by new biological data, of the original network that will serve as our “toy model” and on which we will apply this particular iteration mode.

### Original Mendoza & Alvarez-Buylla Network

This section gives a presentation of the original genetic regulation network of the floral morphogenesis of *Arabidopsis thaliana*, also called *original (Mendoza & Alvarez-Buylla) network* in the sequel. In particular, we focus on its structural and dynamical properties and prove formally why its asymptotic dynamical behaviour can only lead to attractors of period less or equal than 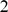, whatever the iteration mode is. Following this, we choose an arbitrary iteration mode with which the study of the dynamics of the network will be carried out and justify this choice with an explanation at the frontier between mathematics and biology.

**Figure 2:**
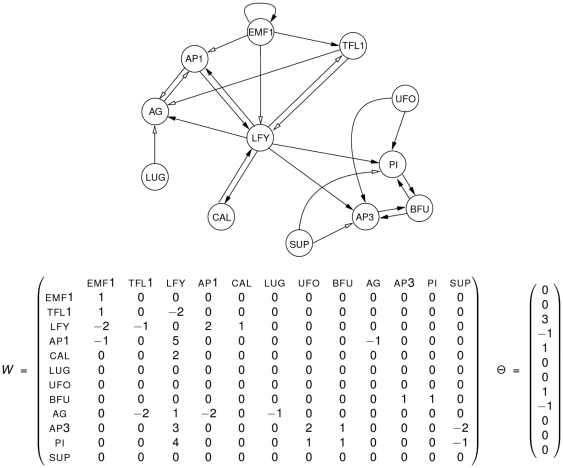
Original Mendoza & Alvarez-Buylla network. Original genetic regulation network modeling the flower morphogenesis of the plant *Arabidopsis thaliana*. Above is pictured the underlying interaction graph. Repressions (resp. activations) are represented by empty arrows (resp. full arrows). Below, the matrix 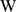 of size 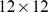 contains the interaction weights between genes and 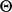 is the thresholds vector.

In [35], the authors isolated twelve genes of the plant *Arabidopsis thaliana* involved in its floral morphogenesis: embryonic flower 1 (emf1), terminal flower 1 (tfl1), leafy (lfy), apetalata 1 (ap1), cauliflower 1 (cal), leunig (lug), unusual floral organs (ufo), agamous (ag), apetalata 3 (ap3), pistillata (pi), superman (sup). A genetic algorithm was used to obtain the interactions between these genes as well as their potentials. From this, Mendoza and Alvarez-Buylla chose to define interaction weights and activation thresholds as signed integers (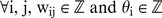). Thus, they proposed a genetic regulation network for the floral morphogenesis of the plant *Arabidopsis thaliana*: the *original Mendoza & Alvarez-Buylla network*. This network, that we denote by 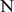, is represented in Figure 2. Its dynamics, revealed by mathematical study, turned out to be particularly close to the reality of the development of the flower.

Considering a specific block-sequential iteration mode, Mendoza and Alvarez-Buylla observed that the configurations of their original network are separated into six attraction basins, all leading to fixed points (cf. Table 1). One interesting point of their study is that, among these six fixed points, four exactly correspond to the four specific tissues of the flower (sepals, petals, carpels and stamens), one corresponds to inflorescence meristematic cells and the last one corresponds to cells that have not yet been seen in nature but that are said to be potentially experimentally induced (see [35] for more details). In the following tables and figures, the names of the six different types of cells are abbreviated: we use *Sep* for sepal, *Pet* for petal, *Car* for carpel, *Sta* for stamen, *Inf* for inflorescence, and *Mut* for the unobserved “cell” (the “mutant” one).

**Table 1:** Attractors of the original Mendoza & Alvarez-Buylla network.

Attractors	Sequential	Parallel	Cell types
Fixed point 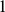	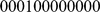	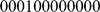	*Sep*
Fixed point 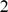	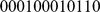	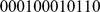	*Pet*
Fixed point 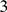	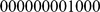	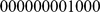	*Car*
Fixed point 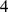	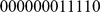	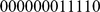	*Sta*
Fixed point 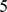	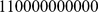	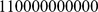	*Inf*
Fixed point 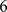	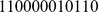	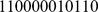	*Mut*
Limit cycle 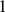	–	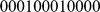 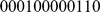	None
Limit cycle 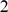	–	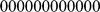 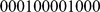	None
Limit cycle 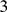	–	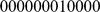 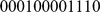	None
Limit cycle 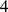	–	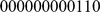 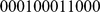	None
Limit cycle 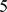	–	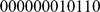 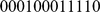	None
Limit cycle 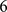	–	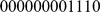 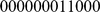	None
Limit cycle 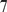	–	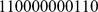 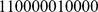	None

Attractors of the original Mendoza & Alvarez-Buylla network dynamics for the sequential and parallel iteration modes and the corresponding cell types. In the descriptions of each configuration, genes are ordered as follows: emf1, tfl1, lfy, ap1, cal, lug, ufo, bfu, ag, ap3, pi, sup.

As mentioned earlier, there are many iteration modes according to which the states of the elements of a network can be updated. When studying the dynamics of deterministic threshold Boolean automata networks modeling real genetic regulation networks, the choice of the iteration mode is far from being trivial for mathematical as well as for biological reasons.

First of all, from the mathematical point of view, studies have shown that different iteration modes can yield significantly different dynamical behaviours for certain threshold Boolean automata networks [5], [8]. The set of threshold Boolean automata networks can be divided into the four following classes according to their robustness against changes of their iteration mode [6], [36], *i.e.*, changes in their asymptotic behaviour depending on the iteration mode:

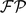
 (for “fixed point”): whatever the iteration mode, every initial configuration of the network evolves towards a fixed point;

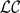
 (for “limit cycles”): whatever the iteration mode, every initial configuration of the network evolves towards a limit cycle;

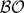
 (for “both”): whatever the iteration mode, some initial configurations evolve towards a fixed point, others evolve towards a limit cycle, so that the asymptotic behaviour of the network always admits at least one fixed point and one limit cycle;

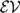
 (for “evolution”): this subset contains the most sensitive networks; depending on the iteration mode, either every initial configuration evolves towards a fixed point, or some of them evolve towards a fixed point and others towards a limit cycle.

As said before, in [35], the original Mendoza & Alvarez-Buylla network was iterated according to a specific block-sequential iteration mode yielding six fixed points. However, it is important to mention that the network can be shown to belong to the class 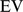 which contains networks whose dynamical robustness appears to be the most complex. Thus, although there are other iteration modes, such as sequential iteration modes, that yield the same asymptotic behaviour as the block-sequential iteration mode chosen by Mendoza and Alvarez-Buylla, there also are other iteration modes, such as the parallel one, for which the evolution of the network leads to the same six fixed points but also to seven limit cycles of period equal to 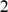 (see Table 1). To understand more precisely the dynamics of the original network, let us recall some theoretical results given by Goles in [29], [37], [38]:

**Theorem 1** *If the interaction matrix* 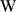 *of a threshold Boolean automata network is symmetric, then the period of its attractors is no more than* 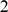 *for any iteration mode*.

**Theorem 2** *If the interaction matrix* 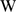 *of a threshold Boolean automata network is symmetric and such that all coefficients in its diagonal are non-negative, then the period of its attractors equals* 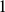 *for any sequential iteration mode*.

We now use both these theorems to explain the dynamics of the original network. Further, we will show how the original Mendoza & Alvarez-Buylla network can be reduced (in terms of arcs) to another whose asymptotic dynamics is equivalent. Then, using this reduced network, we will introduce the notion of *general iteration graph* which will argue for the choice of an arbitrary sequential iteration mode to study the dynamical behaviour of these genetic regulation networks.

**Proposition 1** *For all iteration modes, the dynamics of the original Mendoza & Alvarez-Buylla network converges either towards fixed points or towards limit cycles of period* 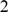.

**Proof** We show that there exists a network 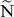, called the *reduced Mendoza & Alvarez-Buylla network*, which is *asymptotically equivalent* to the original network [6], [39], that is, both networks, 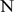 (the original Mendoza & Alvarez-Buylla network) and 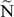, have the same attractors. The dynamics of the network 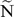 being *governed* by two non-trivial strongly connected symmetric components (all nodes not belonging to these components necessarily end up, within a few steps, having a stable state), theorems 1 and 2 can then be applied.

To build the network 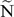, we first derive immediately from the interaction graph of 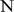 (see Figure 2) that for all block-sequential iteration modes, the states of several nodes become fixed after a few time steps. Indeed, there are no nodes acting on nodes lug, ufo and sup (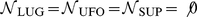) and their activation thresholds all equal 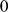 so that as soon as the first update of these nodes:
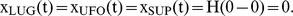
The self-activation of node emf1 and the absence of any other interaction on this node (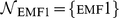) guarantees its state to be constant and equal to its initial value:
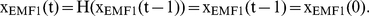
As for node lfy, its activation potential
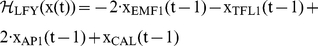
is never greater than 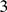. And since 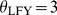, as soon as its first update, the state of node lfy also becomes constantly equal to 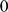:
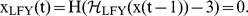
Consider now node cal. The only node acting on its state is node lfy which we have shown to be inactivated after a certain amount of time. Thus:
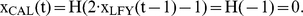
Similarly, the state of node tfl1 depends only on states of nodes that become fixed so that its own state also becomes fixed:
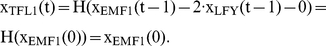
Consequently, the seven genes lug, ufo, sup, emf1, lfy, cal, and tfl1, do not act directly on the dynamics of the network. They serve as a kind of *release mechanism* for the dynamics whose impact vanishes after some time (at most after 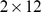 iterations, *i.e.*, after two updates of every node, in the case of a sequential iteration mode). On the contrary, the five other nodes, ag, ap1, pi, ap3, and bfu, play a significant part in the network dynamics.

**Figure 3:**
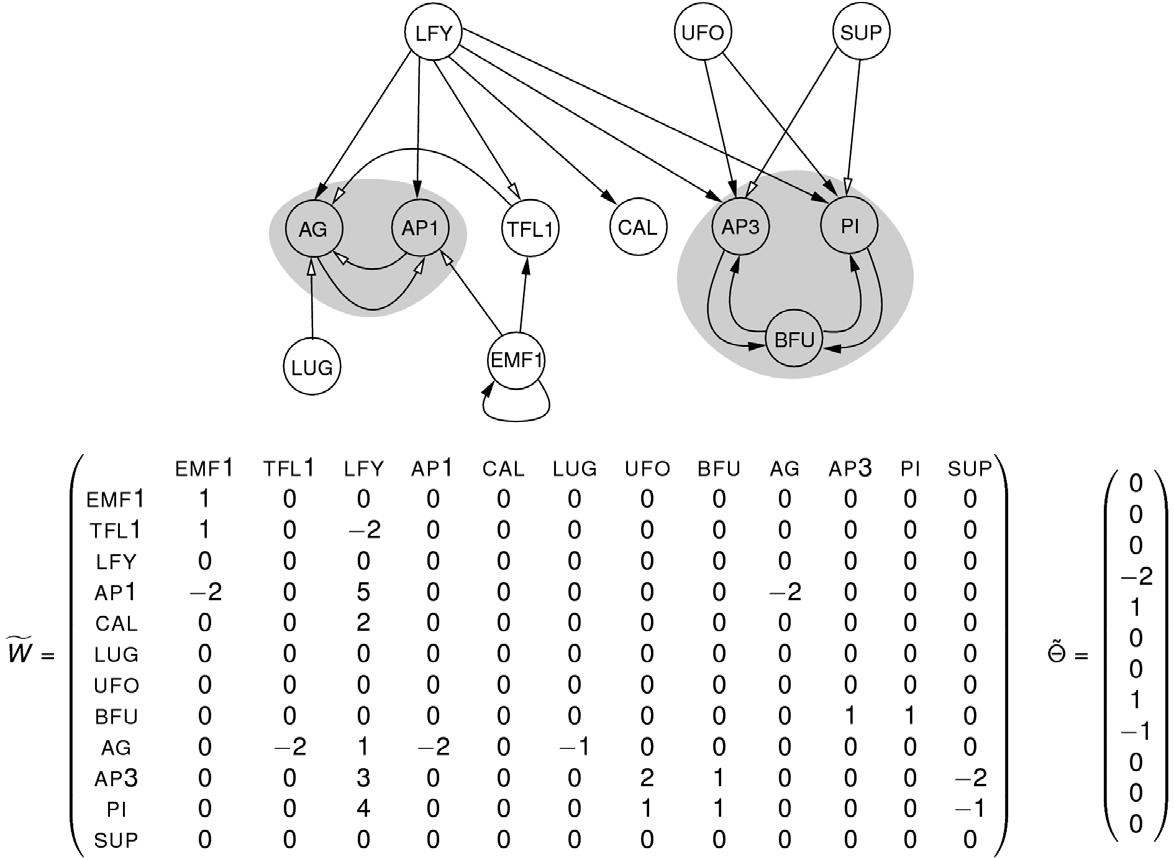
Reduced Mendoza & Alvarez-Buylla network. Genetic regulatory network with two non-trivial strongly connected symmetric components (in grey). The asymptotic dynamics of this network has the same attractors as the original network.

Nodes ag and ap1 interact with one another but otherwise depend only on nodes whose states become fixed so that:
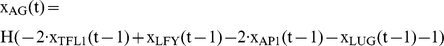


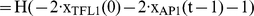
and:
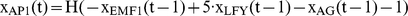


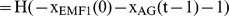


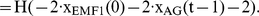
In the last expression above, without changing the local interaction function of ap1, we have doubled all quantities intervening in its interaction potential. This way, we may redefine the activation threshold of ap1 as well as the weight of the interaction that ag has on ap1 so that 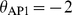 and 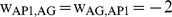. Thus, we may define 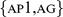 as a strongly connected symmetric component in 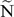. With similar arguments for nodes ap3, pi and bfu, for a big enough 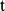, we obtain, :
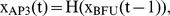


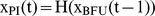
and:
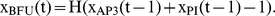
We may thus define 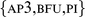 as another strongly connected symmetric component of 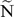. Respecting all constraints found above, we finally construct 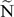 as pictured in Figure 3. Since the dynamics of 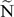 only depends on the nodes of the two non-trivial strongly connected symmetric components 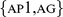 and 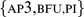, the necessary and sufficient conditions of theorems 1 and 2 hold for 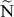 and by its construction, for 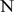 as well.

In the proof of Proposition 1, we have build a simpler version of the original network 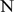 (see Figure 3) which, by construction, has the same asymptotic behaviour as 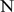. This *reduced Mendoza & Alvarez-Buylla network* 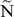 allows an intuitive understanding of the dynamics of 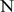 and of the role played by each node in this dynamics. In particular, Proposition 1 explains why sequential dynamics on the Mendoza & Alvarez-Buylla network yield only fixed points whereas the parallel iteration mode yields, as well as these fixed points, some limit cycles of period 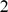. All in all, 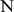 and 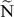 do not, however, behave identically (their behaviour may differ for a few time steps). Thus, in order to stay in adequation with the biological knowledge and keep the same attraction basins (and not just attractors), the rest of our work, and in particular our “toy-model”, is based on the original Mendoza & Alvarez-Buylla network.

**Figure 4:**
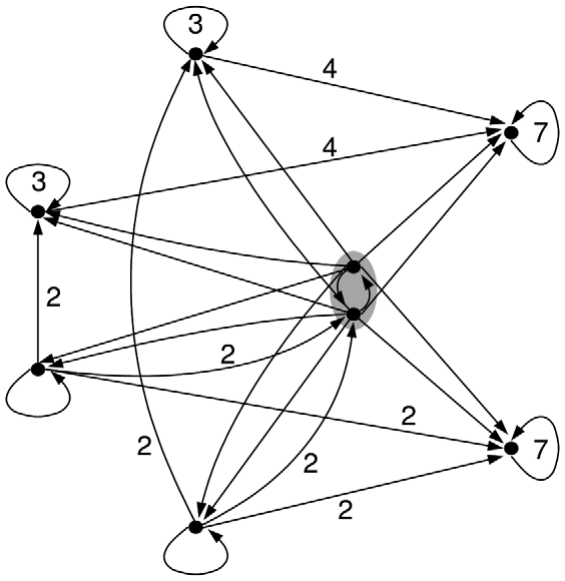
General iteration graph of the strongly connected symmetric component {ap3, bfu, pi}. General iteration graph of the strongly connected symmetric component 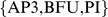 of the reduced Mendoza & Alvarez-Buylla network 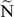 pictured in Figure 3. In this graph, for the sake of clarity, we have represented 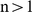 arcs with the same beginning and ending as one unique arc labelled by 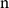. The sub-graph in grey corresponds to a limit cycle of the connected component with the parallel iteration mode. It induces limit cycles 1, 3, 4, 6 and 7 of Table 1. Note that when the state of nodes lfy, ufo and sup is fixed to 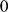 in 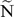 (this always becomes true after a few steps according to the proof of Proposition 1), then the connected component 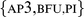 is free to evolve as pictured by this general iteration graph.

**Figure 5:**
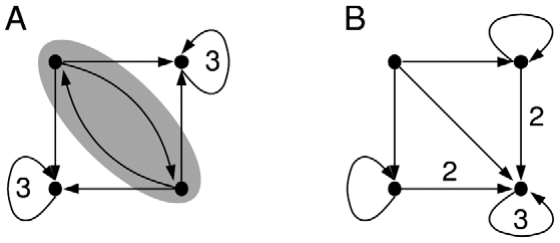
General iteration graph of the strongly connected symmetric component {ap1, ag}. General iteration graph of the strongly connected symmetric component 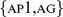 of the reduced Mendoza & Alvarez-Buylla network 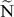 pictured in Figure 3 (a) when the states of nodes emf1 and tfl1 are both fixed to 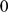 and (b) when they are both fixed to 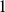. In this graph, for the sake of clarity, we have represented 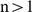 arcs with the same beginning and ending as one unique arc labelled by 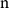. The sub-graph in grey is a limit cycle of the connected component with the parallel iteration mode. It induces limit cycles 2, 3, 4 and 5 of Table 1. Note that when the state of nodes lfy and lug is fixed to 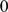 in 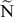 (this always becomes true after a few steps according to the proof of Proposition 1), then the connected component 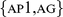 is free to evolve as pictured by one of these two general iteration graphs since no other nodes than emf1 and tfl1 whose states are either both 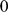 or both 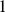 have an influence on them.

As mentioned above, different iteration modes of the original network, may lead to different dynamics. Thus, relying solely on mathematical considerations, one cannot justify reasonably the use of one specific iteration mode rather than another. From the biological point of view, the lack of knowledge concerning the order of gene regulations does not give either any argument allowing to choose appropriately one iteration mode. Nevertheless, biologists' community tends to agree that the probability that the genes involved in a same cellular physiological function evolve in parallel is almost null, particularly in the presence of noise. Thus, one central question is: under what conditions do genes trajectories move across an attraction basin separatrix depending or not on their synchrony [40]–[43]? It does not seem reasonable to think that each gene (and, in particular, its expression) is subjected to a specific genetic biological clock and that all the biological clocks are synchronised, for instance by the dynamics of the chromatin which allows or not the synchronous genes transcription. This dynamics is partly unknown, but has to be compatible with the observed asymptotic behaviour of the genetic networks. For example, in the framework of the floral morphogenesis of *Arabidopsis thaliana*, the parallel iteration mode induces limit cycles (see table 1) that are not actually known to have any biological meaning. Original studies of theoretical biology [30], [31] about discrete models for genetic regulation networks tend to emphasise the use of sequential iteration modes. In the sequel, following in line with Kauffman and Thomas we concentrate on a sequential updating of the network. Although the choice of the sequential iteration mode is arbitrary, we may argue that the principal properties of the network asymptotic dynamics still are captured. To see why, let us first recall that fixed points do not depend on the iteration. As for limit cycles, we claim that not all are meaningful in a sense that we are about to clarify. Let us define the *general* iteration graph associated to a network 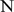 whose interaction graph is 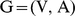. In this general iteration graph, nodes represent configurations and a configuration 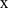 has out-degree 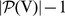 (*i.e.*, the size of the power set of 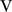 minus 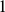 corresponding to the empty set, a *power set* 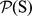 of a set 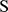 being the set of all subsets of 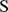). There exists an arc from configuration 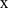 to configuration 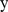 if there is a subset 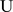 of 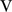 such that, updating all nodes of 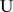 synchronously (and leaving the states of all nodes of 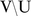 unchanged), 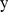 is reached from 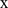 in one step. Note that general iteration graphs generalise the iteration graphs of all block-sequential iteration modes. We have constructed the general iteration graphs of both strongly connected symmetric components 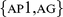 and 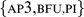 of the reduced Mendoza & Alvarez-Buylla network constructed in the proof of Proposition 1. These graphs are represented in Figures 4 and 5. From them, we derive that the set 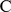 of configurations belonging to the limit cycles observed with the parallel iteration mode (restricted to genes ag, ap1, ap3, pi and bfu), are highly improbable to be reached. Indeed, not only very few arcs of the general iteration graph lead to this set 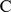 but also, almost all of the outgoing arcs of the configurations 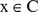 lead to configurations that are not in 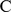. In other words, starting in one particular configuration, the network has very few chances to end in a configuration of 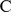 and, if ever it does, the chances are that it will leave it very quickly. Assuming that it is doubtful that real networks such as the one commanding the floral morphogenesis of *Arabidopsis thaliana* may obey infallibly the exact same updating order of their genes, we believe that general iteration graphs do indeed provide evidence of some attractors unlikeliness, as is the case for the limit cycles of the Mendoza & Alvarez-Buylla network observed with the parallel iteration mode. Thus, from now on, we will ignore the possible but improbable limit cycles of the original network and concentrate on its fixed points. Following this choice, Proposition 1 allows us to select arbitrarily one sequential updating mode.

### Variation around the Original Mendoza & Alvarez-Buylla Network: the “Toy Model”

The purpose of this section is to present the main properties of the dynamics of a new genetic regulation network, which is a variation of the original Mendoza & Alvarez-Buylla network that allows to account for the influence of gibberellin (ga). The study of the influence of this boundary element on the dynamical behaviour of the network, the consequences of its absence or presence, will be carried out in the next sections.

**Figure 6:**
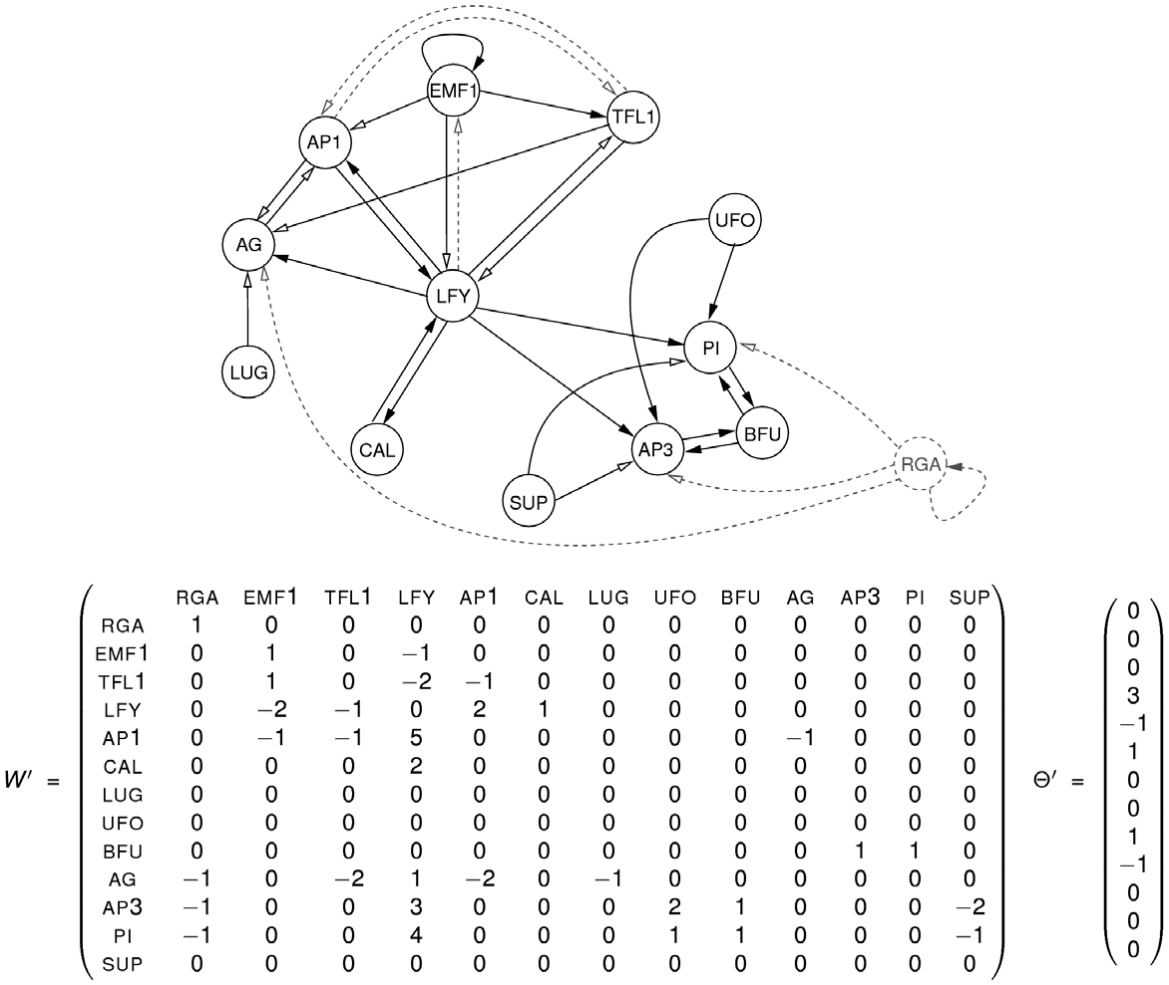
Toy model. Variation of the original Mendoza & Alvarez-Buylla network. This version of the network includes a supplementary node corresponding to gene rga (in dashed lines) to account for the gibberellin's influence on the rest of the network. Above is pictured the interaction graph of this network. Repressions (resp. activations) are represented by empty arrows (resp. full arrows). Nodes and interactions added to the original network are indicated in dashed grey. The matrix 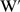 of size 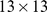 contains the interaction weights. 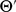 is the activation thresholds vector.

We build the new network 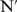 from the original network described in [35]. First, we add to it all the non hypothetical interactions presented in [44] (without adding any new vertices). More precisely, we add the three following inhibitions, assuming that their interaction weight is minimal: lfy 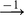 emf1, ap1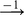 tfl1 and tfl1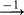 ap1. In [19], Yu et al. explain that gibberellin reduces the stability of a specific protein, which is the product of a gene called repressor of ga (rga). More precisely, they report that “ga promotes the expression of floral homeotic genes by antagonizing the effects of della proteins, thereby allowing continued flower development.” By the terms “floral homeotic genes”, the authors mean ag, ap3 and pi; by “della proteins”, they refer in particular to rga. In the context of threshold Boolean automata networks, there are two ways of interpreting this result. The first one is to consider that rga is a repressor of ag, ap3 and pi and ga is a repressor of rga. The second one is to consider that ga is an activator of ag, ap3 and pi and rga is a repressor of ga. It is important to note that the instantiation of these two interpretations leads to the same results. Moreover, Yu et al. mention that, according to recent studies, ga overcomes the function of della repressors by inducing degradation of their proteins. For sake of coherence with this statement, we have chosen to instantiate the first interpretation, in which rga inhibits the expression of ag, ap3 and pi and ga inhibits the expression of rga. Subsequently, we add to the network one node representing rga and the four following interactions: rga 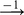 ag, rga 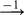 ap3, rga 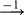 pi and rga 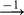 rga. An illustration of this new network is given in Figure 6. By definition of threshold automata networks, what emerges from the structure of this new network is that: when gibberellin is present, the state of gene rga is fixed to 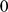 and, when it is absent, its state can be either 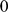 or 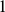. Note that we add the fourth interaction, the rga self-activation, to convey the ability rga has to maintain itself activated in the absence of gibberellin. The values we chose to give to the weights of the new interactions are voluntarily minimal (*i.e.*, their absolute value is equal to 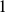) because of our wish to focus more on the structure of the network than on the specific values which have little chance to be realistic anyway. Let us eventually point out that gene rga is a boundary node of the network according to the mathematical definition given earlier.

In order to make easier the understanding, in the notation of an arbitrary configuration 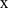, we will isolate node rga as follows:
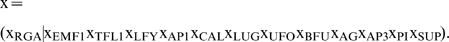



Now that the major elements have been described and defined, we are going to focus on the influence that the peripheral gene rga has on the dynamics of the regulation network. This study is going to show why the absence of gibberellin impedes the normal development of the flower and conversely why its presence promotes it and thereby guarantees its reproduction. As said before, this study is performed on a specific example of real genetic regulation network but the reader has to keep in mind that the method provided is theoretically applicable to understand or explain the influence of boundary conditions on any discrete dynamical system, with a particular relevance in the context of genetic regulation networks whose asymptotic dynamical behaviour leads to attractors corresponding to cellular tissues. We will discuss later that, in practice, using this approach and drawing some significant results from it is, of course, limited by the inherent exponential computational complexity of any discrete dynamical system. In the sequel, we are going to emphasise that attraction basins are relevant gauges to highlight the influence of boundary conditions on discrete dynamical systems. More precisely, they give good indications on the dynamical properties of a system. Thus, as our objective is to understand and highlight the mathematical properties induced by the absence/presence of gibberellin on the floral morphogenesis of *Arabidopsis thaliana*, we have chosen to show the differences between the dynamical behaviour of the genetic regulation network when the state of the boundary gene rga can change (absence of gibberellin) and when it is fixed to 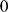 (presence of gibberellin).

For reasons given above, the following study will be based on the sequential iteration mode (whose induced asymptotic dynamics on the network is compared in Table 7 at page 26 to that of the block-sequential iteration mode chosen by Mendoza & Alvarez-Buylla in [35]) defined by the following ordered partition of the set of nodes of our toy network 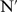:
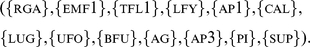
Now, considering this iteration mode, when the state of the peripheral node rga is equal to 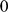, obviously, the network 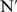 evolves towards the same six fixed points as the original network 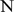. When, the state of rga equals 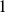, however, we obtain two supplementary fixed points. Since the six different cell lineages are defined independently of the state of the rga gene, we can identify one of these two fixed points with the sepal lineage and the other with the inflorescence lineage (see Table 2). Then, merging attractors as well as attraction basins corresponding to identical cellular types will allow us to simplify our study by reducing the number of these sets from eight to six. More precisely, we will assume that the attraction basin of the sepal lineage is the union of the attraction basins of the fixed points 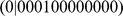 and 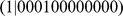 and the attraction basin of the inflorescence cells is the union of the attraction basins of the fixed points 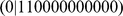 and 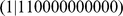. Hence, in both the cases of the absence and the presence of gibberellin, we retrieve exactly six attraction basins and attractors. This will ease comparisons between the results obtained in both cases.

**Table 2:** Fixed points of the toy model 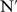 and the corresponding floral tissues of *Arabidopsis thaliana*.

Mathematical fixed point	Biological tissue
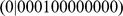	*Sep*
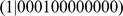	*Sep*
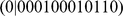	*Pet*
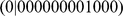	*Car*
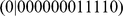	*Sta*
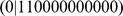	*Inf*
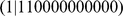	*Inf*
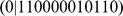	*Mut*

In the following section, we propose to concentrate on the impact that gibberellin has on the attraction basins of the genetic regulation network 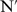. To do this, we focus on three different attraction basin properties: their absolute and relative sizes, their relative distances (this notion will be defined later) and their robustness against stochastic state perturbations. Before detailing our scientific generic approach and presenting the major results obtained concerning the influence of gibberellin on the floral morphogenesis of *Arabidopsis thaliana*, let us give some intuition about the relevance of these three attraction basin properties. First of all, because we do not want to introduce any bias in favour or in disfavour of any initial configuration, our study is set on the hypothesis that any configuration has same chances to be chosen initially, *i.e.*, the random choice of initial configurations is done uniformly. Thus, conclusions that are drawn in the sequel may be considered true even if there are no privileged initial conditions, that is, in particular, even if configurations of the floral basins are as likely as any other. Said otherwise, our results give an information on the tendencies of the system evolution as long as initial conditions do not present a strong bias in favour of the mutant/inflorescence basins. Considering that nature does seem to ease the development of the floral basins, we believe that the hypothesis of uniformity does convey some reasonable and meaningful information.

Let us now consider an arbitrary attraction basin 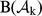 of a discrete dynamical system. The *relative size* of 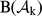 yields the probability to choose, randomly and uniformly, an initial configuration in this attraction basin. Thus, if the discrete dynamical system studied is a genetic regulation network and 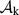 is an attractor corresponding to a real cellular type, then the relative size of 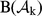 gives an indication on the probability that the physiological function represented by the network is to create a cell of the lineage corresponding to 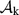. *Relative distances* between attraction basins give an insight of the probabilities for an initial configuration belonging to an attraction basin 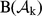, once perturbed, to become a configuration of another attraction basin 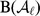. Let 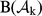, 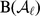 and 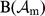 be three different attraction basins. If the relative distance from 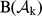 to 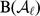 is smaller than the one from 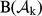 to 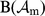, small perturbations on an arbitrary configuration belonging to 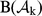 have more chances to change it into a new configuration belonging to 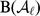 than into one belonging to 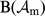. The final step of our approach is to go further studying rigorously the robustness of the dynamical behaviour of a discrete dynamical system against *stochastic state perturbations* depending on a state perturbation rate denoted by 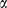 (using attraction basins as gauges). In our toy model, this last step allows us to prove that the presence of gibberellin significantly increases the probabilities for the flower of *Arabidopsis thaliana* to develop normally and, further, that its presence is a necessary condition for floral morphogenesis.

## Results

### Sizes and Relative Distances

In this section, we first detail the results on the influence of gibberellin drawn from the study of the relative sizes of the attraction basins of the network 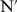. Then, we focus on what can be learnt from an analysis of relative distances between all ordered couples of attraction basins. Let us recall that all the results of this section, on the attraction basins sizes as well as on their relative distances, are based on the hypothesis of uniformity discussed previously.

### Absolute and Relative Sizes of Attraction Basins

Let us first define formally the notions of absolute and relative sizes of an attraction basin.

**Definition 1** *The absolute size of an attraction basin* 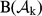 *of a discrete dynamical system* 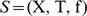 *is the cardinal of* 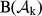.

**Figure 7:**
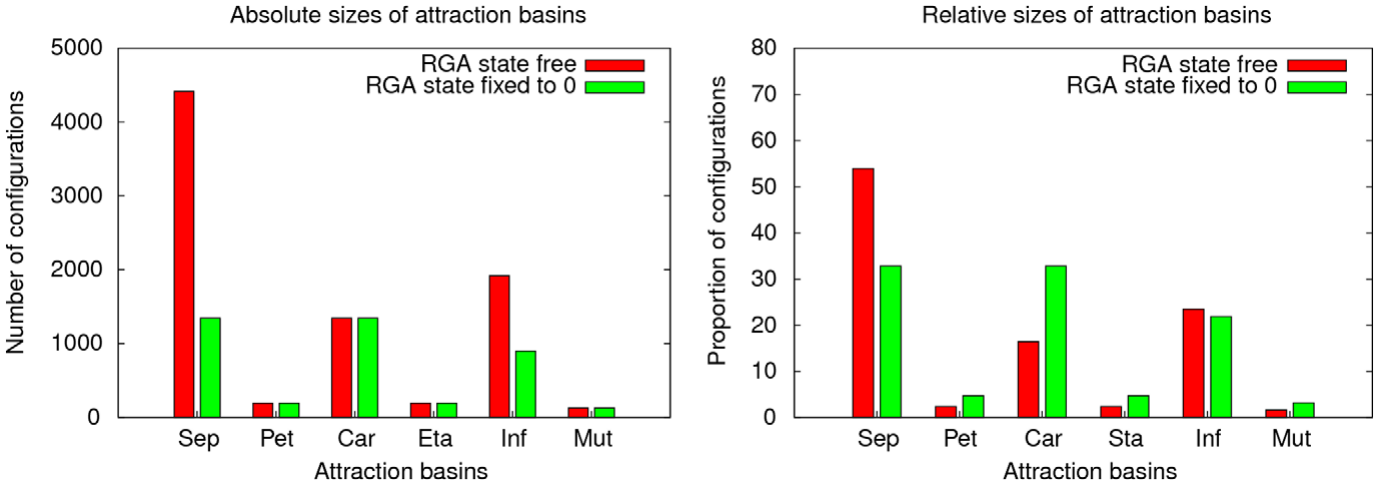
Attraction basins sizes. Histograms representing the absolute sizes (left panel) and the relative sizes (right panel) of the attraction basins of the genetic regulation network of the floral morphogenesis of the plant *Arabidopsis thaliana*, depending on the absence or presence of gibberellin.

**Definition 2** *The relative size* 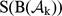 *of an attraction basin* 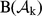 *of a discrete dynamical system* 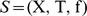 *is*:
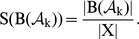



Our toy network 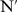 is composed of 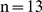 nodes. Its total number of possible configurations is therefore equal to 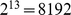 when the state of gene rga is free (absence of gibberellin) and to half of that, 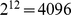, when the state of rga is fixed to 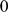 (presence of gibberellin). Figure 7 presents in two histograms, the absolute and relative sizes of every biological attraction basin of the network.

Comparing the absolute sizes of attraction basins, we derive that the 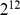 configurations in which the state of rga is 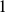 (this can only be observed when there is no gibberellin flow) are only distributed into two attraction basins, that corresponding to the sepal lineage and that corresponding to the inflorescence lineage: three quarters of the configurations in which the rga state is 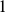 are sepal configurations and one quarter of them are inflorescence configurations. Computations that compared configurations contained in each attraction basin according to the absence/presence of the hormone allowed us to detail the contents of the *Sep* and *Inf* basins when there is no hormone. On one hand, the *Sep* basin in absence of gibberellin contains all the 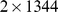 configurations 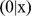 (in which 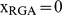) and 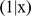 (in which 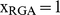) where 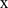 is one of the 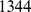 configurations contained in the *Sep* basin when there *is* some hormone. It also contains all the 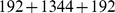 configurations 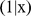 where 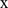 is a configuration belonging to one of the floral basins (*Pet*, *Car*, *Sta*) in presence of gibberellin. On the other hand the *Inf* basin in absence of gibberellin contains all configurations 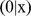 and 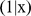 where 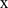 is a configuration belonging to the *Inf* basin in presence of the hormone, as well as all configurations 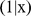 where 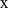 is a configuration belonging to the *Mut* basin in presence of the hormone. These observations are confirmed by the sizes of each attraction basin in the the case where the rga node is free to take any state, in the case where its state is fixed to 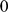, and in the case where it is fixed to 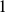. These sizes are given in Table 3.

**Table 3:** Absolute sizes of the attraction basins in absence/presence of gibberellin.

	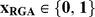	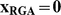	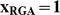
*Sep*	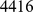	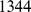	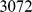
*Pet*	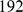	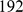	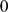
*Car*	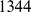	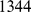	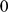
*Sta*	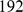	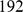	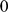
*Inf*	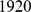	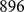	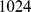
*Mut*	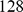	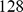	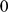

Absolute sizes of the attraction basins when the state of rga is free (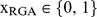), is null (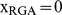) and is equal to one (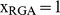).

Attraction basins corresponding to other cell lineages have the same absolute size whether gibberellin is present or not. This explains why their *relative* sizes are twice as big when rga is fixed to 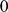 than when it can take any state. Now, as discussed above, in the field of genetic regulation networks, the relevance of the relative sizes of attraction basins lies in the fact that they convey how likely the system is to create a cellular tissue corresponding to a specific attractor. Thus, the likeliness of configurations corresponding to petal, stamen, carpel and mutant tissues is doubled when rga is fixed to 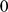. The biological meaning of this is that in presence of gibberellin, the plant can create twice more petals, stamens and carpels. In particular, the presence of gibberellin brings 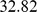
% of the configurations towards the biological attractor of carpels, while only 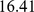
% of the configurations evolve towards this attractor when gibberellin is absent. This is important because carpels are the female genital organs of the plant. Thus, they are necessary for its floral development and their presence guarantees the ability of the plant to reproduce itself. Furthermore, let us point out that the absence of gibberellin also creates an important disequilibrium between the sizes of attraction basins at the expense of most floral tissues (sepals, petals, carpels and stamens): when the boundary node rga can change its state, the differences in the proportions of the different attraction basins is accentuated. 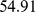
% (resp. 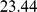
%) of the initial configurations lead to sepal cells (resp. inflorescence cells) whereas only almost 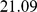
% of them lead to petal, carpel and stamen cells. Forcing the inhibition of the boundary significantly reduces this disequilibrium. Biologically, this means that the inhibition of rga forced by the flow of gibberellin (ga) significantly improves the chances that the plant has to develop normally. This was experimentally observed in [19].

### Relative Distances Between Attraction Basins

Another pertinent measure of the influence of gibberellin on the dynamical behaviour of the floral morphogenesis of *Arabidopsis thaliana* is given, we believe, by the differences in the global distances separating attraction basins. When we choose arbitrarily a cell in the flower of *Arabidopsis thaliana*, although it contains the same genetic material as the other cells, it has specialised itself to code for a specific physiological function. This function may correspond to one of the four floral tissues, sepal, petal, carpel or stamen. However, it is possible that specific biological events or elements can perturb this cell into making it specialise to code for a different physiological function. We may reasonably suppose that when this happens, the cell is more likely to adopt one of the cellular types that are the closest, from a biological point of view, to its original cellular type. Hence, in this section, we are going to study distances between attraction basins in order to analyse on our toy model the impact that random perturbations have on the evolution of the system or the *fate* of a cell.

We use a classical notion of distance between configurations: the Hamming distance as defined below.

**Definition 3** *Let* 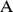 *be an alphabet and* 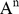 *be the set of words of length* 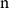 *with letters in* 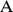. *The Hamming distance* 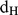 *between any two words* 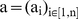 *and* 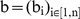 *of* 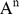 *is the number of letters that differ in words* 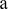 *and* 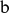, *i.e.*, 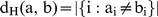.

In order to have a metric between attraction basins, we use the *modified Hausdorff distance* between sets that was introduced in [45]. Its basic metric is the Hamming distance:

**Definition 4** *Let* 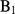 *and* 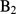 *be two sets of words of same length and defined on the same alphabet*. *The relative distance from set* 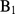 *to set* 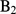 *is defined by the arithmetic average*, *over all words* 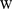 *of* 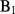, *of the minimal Hamming distance between* 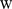 *and all words in* 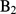. *Formally*:
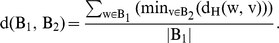



Of course, since in particular 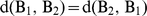 is not necessarily true, strictly speaking, 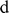 is not a distance from the mathematical point of view (note that the same misuse of language exists in the graphs theory about the notion of distance between two vertices in a directed graph). Consequently, we have decided to use the term *relative* to qualify this specific notion of distance. Exhaustively, we computed relative distances between each ordered couple of attraction basins. The results obtained are given in Table 4 below.

**Table 4:** Relative distances separating attraction basins.

	*Sep*	*Pet*	*Car*	*Sta*	*Inf*	*Mut*
*Sep*	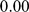	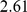	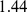	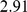	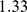	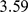
*Pet*	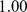	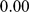	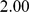	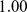	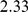	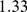
*Car*	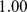	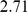	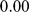	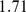	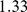	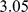
*Sta*	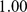	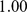	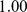	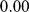	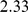	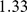
*Inf*	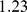	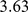	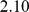	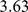	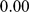	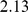
*Mut*	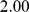	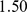	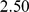	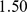	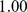	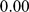
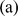						

Relative distances separating attraction basins, 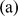 when the state of the boundary node rga is free (absence of gibberellin) and 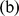 when its state is fixed to 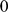 (presence of gibberellin). The distance from the inflorescence attraction basin to the stamen attraction basin, for instance, is read at line *Inf* and column *Sta*. It is 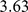 or 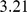 depending on the state of rga being fixed or not.

As we discussed earlier, sepal and inflorescence attraction basins are the only ones whose absolute sizes are subjected to variations depending on the presence/absence of gibberellin. Therefore, the only relative distances that could change according to the presence/absence of gibberellin are distances *from* or *to* one of these two attraction basins. In reality, distances from petal, carpel and stamen attraction basins to any other attraction basin do not undergo any change, neither do any of the distances *to* the inflorescence basin (see Table 4). On the contrary, distances *from* attraction basins sepal and inflorescence to others do differ significantly. Let us focus on them.

Consider first the sepal attraction basin. When the state of rga is fixed to 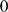 (Table 4 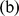), its distances to other basins are notably smaller than when it is not (Table 4 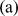), especially 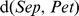, 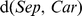 and 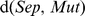. Nevertheless, the order of these relative distances remains the same: carpel basin stays the closest basin to the sepal basin and mutant basin stays the furthest. This means, in particular, that in both cases, the probability that state perturbations acting on initial configurations of the sepal basin will lead to configurations belonging to the carpel basin remains high (this will be confirmed in the next section by an analysis on stochastic state perturbations). We can also observe that the presence of gibberellin tends to bring closer together the sepal basin and both the petal and carpel basins: their relative distances fall respectively from 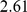 down to 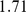 and from 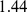 down to 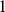 when gibberellin appears. This is meaningful because petals and carpels are cellular types that are very important to the plant development: petals attract insects that can transport pollen, and carpels allow reproduction. Similar results may be derived by focusing on the inflorescence basin. Indeed, the presence of gibberellin significantly reduces the distances from inflorescence configurations to carpel and petal configurations. The distance that is reduced the most is the distance to the carpel basin, which, again, confirms the importance of the hormone influence on the floral morphogenesis of this plant.

To go further, instead of considering *average* relative distances, we are now going to look at the probability distributions of relative distances, that is, the proportion of configurations 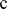 in 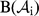 whose relative distances to another attraction basin 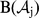 is 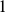, 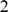, etc. Tables 5 and 6 illustrate these distributions when the attraction basin of origin 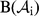 is, respectively, the sepal basin and the inflorescence basin. More precisely, they present numerically the proportions of configurations of the sepal and the inflorescence attraction basins, respectively, that are at a given distance to other attraction basins, according to whether the state of gene rga is fixed to 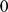 or not.

**Table 5:** Relative distances from the sepal attraction basin.

	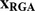	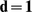	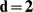	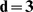	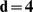	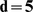	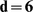
*Pet*	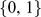	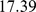	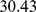	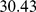	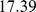	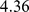	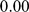
	0	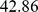	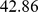	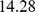	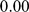	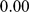	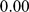
*Car*	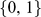	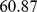	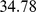	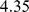	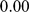	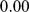	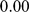
	0	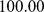	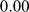	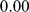	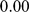	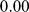	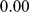
*Sta*	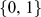	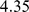	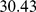	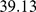	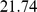	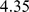	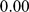
	0	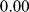	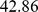	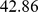	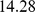	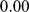	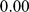
*Inf*	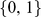	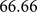	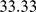	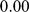	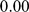	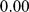	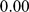
	0	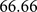	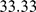	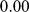	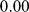	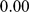	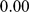
*Mut*	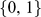	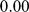	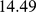	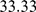	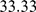	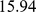	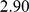
	0	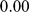	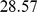	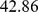	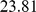	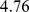	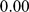

Probability distributions (in percentages) of the relative distances separating configurations of the sepal attraction basin from configurations of other attraction basins.

**Table 6:** Relative distances from inflorescence attraction basin.

	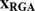	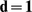	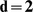	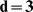	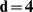	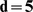	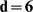
*Sep*	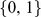	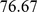	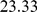	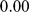	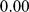	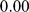	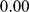
	0	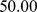	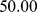	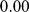	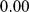	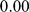	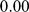
*Pet*	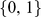	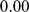	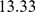	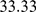	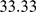	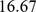	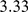
	0	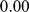	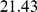	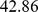	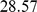	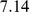	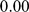
*Car*	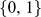	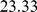	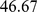	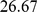	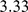	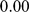	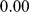
	0	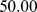	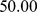	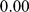	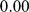	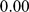	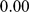
*Sta*	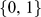	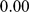	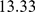	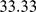	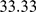	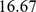	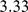
	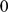	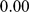	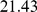	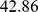	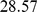	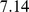	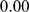
*Mut*	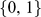	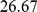	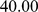	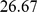	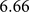	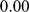	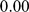
	0	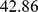	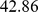	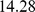	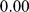	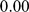	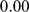

Probability distributions (in percentages) of the relative distances separating configurations of the inflorescence attraction basin from configurations of other attraction basins.

From Tables 5 and 6, one may note that fixing the state of rga to 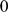 has several effects on these distances. First, we have seen earlier that configurations in which the state of rga is 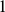 do not belong to the attraction basins petal, carpel, stamen and mutant (they all belong either to the sepal basin or to the inflorescence basin). As a result, in absence of gibberellin, every such configuration is at least at distance 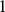 to any of these four attraction basins. Computations have shown that approximately 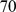
% (resp. 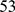
%) of the configurations of sepal (resp. inflorescence) basin when there is no gibberellin are configurations in which the state of rga is 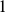. Since the sepal and inflorescence basins contain such significant proportions of these configurations, we ignore one unit reductions of maximal relative distances caused by the hormone presence. Now, let us note that while distances of the sepal basin to the inflorescence basin are not influenced at all by the presence of gibberellin (rga state is fixed to 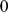), maximal distances from sepal configurations to the petal and to the carpel basins both loose two units when gibberellin appears. Moreover, the proportion of *Sep* configurations at distance 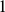 of the *Pet* basin (resp. the *Car* basin) undergoes a significant increase from 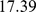
% (resp. 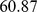
%) to 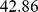
% (resp. 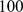
%) when the hormone becomes present. These observations convey the impact that gibberellin has on the sepal attraction basin: it draws it much closer to the petal and carpel basins.

Let us now concentrate on Table 6. Gibberellin tends to reduce distances from the inflorescence basin to the others, with the exception of the sepal basin. In particular, we may observe that the only maximal distances that undergo a two unit reduction are the maximal distances of *Inf* configurations to the *Car* basin. As for the sepal basin, when there is no gibberellin there are 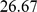
% more configurations of the *Inf* basin that are at distance 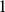 from the sepal basin than when there is gibberellin. These 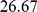
% supplementary configurations are in reality configurations in which the state of rga is 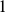. Thus, when gibberellin appears and the state of rga becomes 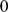 and these configurations disappear. This significant difference of proportion is echoed on the probability distribution of relative distance separating *Inf* configurations from *Car* configurations. Indeed, there are 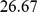
% more *Inf* configurations at distance 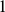 of the *Car* basin in presence of gibberellin (the proportion of these configurations rises from 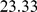
% to 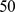
% when the hormone appears).

These results constitute another important step that emphasises the role of gibberellin on the floral morphogenesis of the plant *Arabidopsis thaliana*. Indeed, when there is no gibberellin and the state of gene rga is free, the sepal and inflorescence attraction basins are appreciably further away from the floral basins. The presence of gibberellin, however, eases transfers of configurations towards floral basins. To go further in these lines, the next section introduces an algorithm to study the robustness of attraction basins against stochastic state disturbances.

### Influence of Stochastic State Perturbations on Attraction Basins

In this section, we present a solution that allows to measure the propensity of a regulation network, and more generally of any discrete dynamical system, to change its dynamical behaviour when it is subjected to state perturbations (under the hypothesis of uniformity discussed on page 10). More precisely, we study here how perturbations of the states of genes exerted on an initial configuration of the network can transform it such that the new configuration belongs to an attraction basin different from that of the initial configuration. Although the type of perturbation is different, the general idea is inspired by the work of Fatès and Morvan on the robustness of cellular automata against asynchronism [7], [10].

We first introduce the algorithm that we implemented to study exhaustively the effects of stochastic state perturbations on the attraction basins of a discrete dynamical system with a small number of elements. This algorithm gives the characteristic polynomials of the probabilities that perturbed trajectories remain in their attraction basins of origin, according to a state perturbation rate 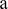. Then, in order to show the pertinence of our algorithm in the field of Systems Biology, we present results obtained by applying it to our toy model, still considering both kinds of boundary conditions as it has been done in the previous section.

### Algorithm

Let 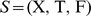 be an arbitrary discrete dynamical system and let 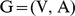 be its interaction graph. Here, we suppose that the system is Boolean, *i.e.*, the states of elements of the system belong to 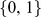 and 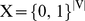. However, the analysis unfolded in this section and the algorithm that ensues can, conceptually speaking, easily and naturally be extended to systems whose elements can have more than just two states. Let us also assume that the dynamical behaviour of 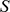 is perfectly known, *i.e.* all the trajectories, the 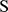 attractors and the 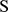 attraction basins of the system have, for instance, already been extracted by a simulation of the evolution over time of the 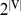 possible initial configurations. We want to measure the influence of stochastic state perturbations on the attraction basins of the dynamical system 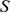. We introduce a stochastic parameter, the *stochastic state perturbation rate*, denoted by 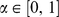. This parameter is the uniform probability of a node of the system to change its state.

**Figure 8:**
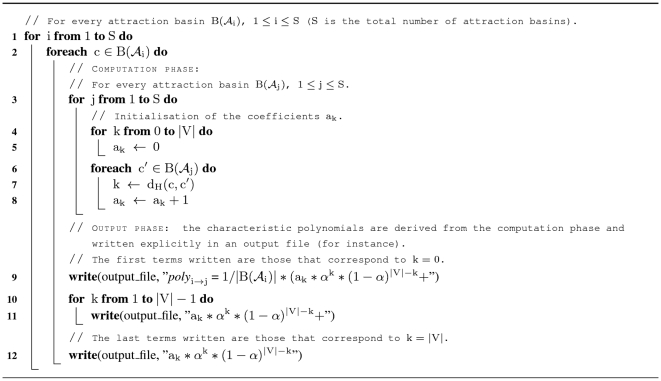
Algorithm. Algorithm that writes in explicit form the characteristic polynomials (with indeterminate the state perturbation rate 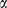) of the probabilities of passage from any attraction basin to any other.

Now, let 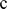 and 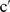 be two configurations of the system. The probability that 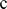 turns into 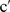, because of a random state perturbation with rate 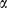, equals the probability that the perturbation changes exactly the states of the 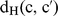 nodes 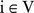 such that 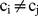 and leaves all others unchanged. This probability depends on the rate 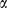:
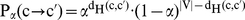
Given two attraction basins 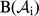 and 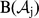, we can derive from the expression above the probability for any arbitrary configuration 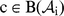 to transform into any configuration 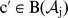:
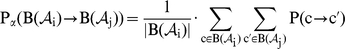


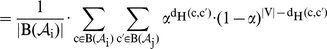


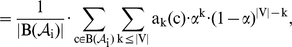
where 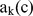 is the number of configurations 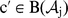 that are at distance 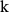 of 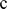, *i.e.*, such that 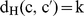. We call this probability the *probability of passage* from one attraction basin to another. As discussed above, if the discrete dynamical system 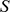 is sufficiently small, thanks to an attractor extraction phase, all quantities involved in the expression of 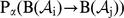, excepted the unknown stochastic state perturbation rate 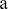, can be obtained. Thus, probabilities of passage 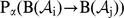 can be seen as polynomials whose indeterminates are the rate 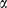. These polynomials are the characteristic polynomials of the probabilities of passage. The purpose of the algorithm of Figure 8 is precisely to compute all of these polynomials (*i.e.*, all the expressions 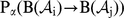 as a function of 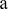 for all 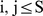, 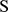 still being the total number of attractors and of attraction basins).

In complexity theory, one notes: 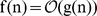 if 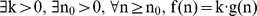. Of course, the complexity of the algorithm of Figure 8 is very high because, for all ordered couples of attraction basins 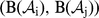, it has to run through all configurations belonging to both basins. More precisely, lines 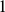 and 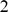 define two *for-loops* that go through all configurations 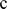 of all attraction basins 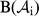. And nested inside these two for-loops, are two others starting at lines 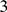 and 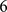 that also go through all configurations 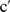 of all attraction basins 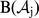. The time complexity inherent to each of these couples of loops is 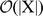. Since 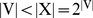, the time complexity of lines 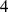 and 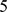 and of lines 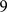 to 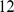 is negligible. Thus, we find that the total time complexity of algorithm of Figure 8 is:
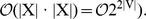
This time complexity, exponential in the size of the input, means that the algorithm can be used to compute the characteristic polynomials of probabilities of passage for systems 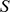 that contain only a small number of elements. In other words, 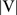 must be sufficiently small (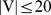, for instance) so that 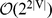 does not represent too long a running time for the algorithm). Let us insist on the fact that, from the point of view of theoretical computer science, the Boolean aspect of our mathematical model is not of importance with respect to the time complexity of the algorithm. Indeed, the algorithm is exponential depending on 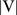 (but quadratic depending on 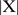). Nevertheless, limitations of the algorithm of Figure 8 due to its time complexity can however be bypassed by settling for relevant approximations of the characteristic polynomials. For instance, a Monte-Carlo method could be included or, as we will discuss later, the number of initial configurations 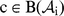 that are run through by the algorithm could be reduced. The effect of this would be to cut down significantly the time complexity without loosing any biological relevance.

### Application to our Toy Model

**Figure 9:**
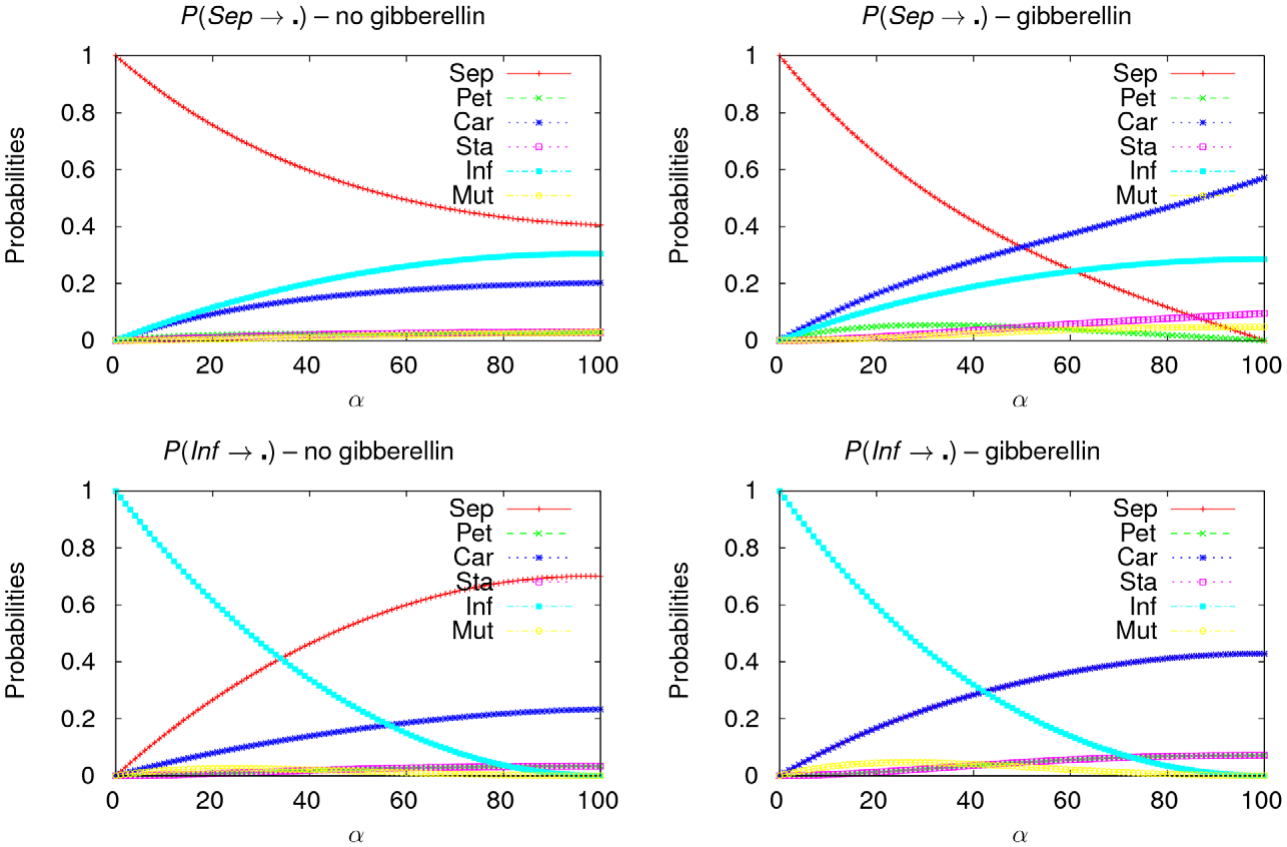
Characteristic polynomials. Curves of the characteristic polynomials with indeterminate the state perturbation rate 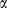 (in percents). The top panels (resp. the bottom panels) plot the curves of the characteristic polynomials of the probabilities of passage from the sepal (resp. inflorescence) attraction basin to another attraction basin when the state of rga is free (left panel) and when it is fixed to 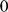 by the presence of gibberellin (right panel). Every panel plots six curves, one for each ordered couple of attraction basins. In the bottom panels, several curves are superimposed: the curves of the characteristic polynomials corresponding to the probabilities to become either a *Pet* configuration or a *Sta* configuration in the left panel as well as the curves of the characteristic polynomials corresponding to the probabilities to become either a *Sep* configuration or a *Car* configuration in the right panel.

In this section, we study the robustness of these two basins against stochastic state perturbations. Figure 9 plots the characteristic polynomials (outputted by the algorithm of Figure 8) of the probabilities for configurations to go from one attraction basin that is either sepal or inflorescence, to any other attraction basin.

Let us first concentrate on the sepal case (top two panels of Figure 9). Consider the probabilities that perturbed configurations stay in the sepal attraction basin. In the top left panel of Figure 9, we can see that, for all values of the perturbation rate 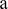, this probability is very high when the state of rga is free. Even when the perturbation causes all nodes to change state (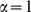) approximately 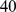
% of all sepal configurations remain sepal configurations. This result illustrates those obtained by experimentation in [18], [19] highlighting that the floral development process of *Arabidopsis thaliana* is hindered in absence of gibberellin. On the contrary, when the state of rga is forced to 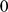 by the presence of gibberellin and when 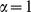, *all* sepal configurations turn into configurations belonging to different attraction basins. Moreover, the probability of *Sep* configurations to become *Inf* configurations is high and increases with 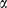. The presence of gibberellin, however, does not change the corresponding characteristic polynomial 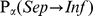. This confirms the observation made earlier concerning the relative distance from *Sep* to *Inf* not changing according to the presence/absence of gibberellin (see fourth panel of Figure 5).

On the contrary, gibberellin does impact on the other characteristic polynomials. Earlier, we saw that gibberellin reduces relative distances between sepal configurations and floral configurations. Figure 9 confirms this. From the biological point of view, some perturbations are bound to occur and act over time on the dynamical behaviour of a genetic regulatory network, provided these perturbations are not too substantial. Therefore, we assume that a state perturbation is “reasonable” when the stochastic state perturbation rate is not greater than 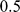. Thus, in presence of reasonable state perturbations, mean probabilities for a configuration of the *Sep* basin to turn into a configuration of a floral attraction basin rise when gibberellin appears. This is particularly true for the carpel basin. In the same lines, when the state of rga is free, the characteristic polynomials of the probabilities of passage from the *Sep* basin to the basins *Pet* and *Sta* are all almost identical and very slightly increasing. Their maximal value which is reached when 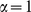 is very low (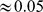). This is not in favour of the natural floral morphogenesis of the plant. In presence of gibberellin, however, at reasonable values of 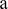, configurations which do not turn into *Car* or *Inf* configurations have significant chances to turn into *Pet* configurations (which also are very important since they represent tissues that attract insects *i.e.*, the most intensive media for bringing pollen). For all these reasons we may again conclude that the presence of gibberellin is favourable to the development of floral tissues.

As for the inflorescence case (bottom two panels of Figure 9), we may first observe that the presence of gibberellin has little impact on the probabilities of configurations subjected to stochastic state perturbations to stay in the *Inf* basin. These probabilities remain high for reasonable values of the rate 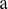. On the contrary, the probabilities of configurations to *leave* the *Inf* basin do change according to whether gibberellin is present or not. From the bottom left panel we derive that a great part of the *Inf* configurations tends to be transformed into *Sep* configurations when there is no hormone but when there is, the probability of passage from *Inf* to *Sep* falls down noticeably. And, this seems to happen for the benefit of the other floral attraction basins *Pet*, *Car* and *Sta* whose corresponding characteristic polynomials increase. So again, deriving information from the characteristic polynomials leads to the conclusion that gibberellin tends to re-equilibrate the likeliness of attraction basins for the benefit of basins corresponding to floral tissues.

Another biologically meaningful way to take into account the influence of stochastic state perturbations is to consider the evolutionary process inherent to the plant morphogenesis [46], [47]. Indeed, assuming that the evolutionary process can be modeled by layered graph, where layers correspond to specific dynamical sub-systems at different stages of the morphogenesis, it is relevant to restrict perturbation only to states belonging to attractors. We implemented another algorithm that induces a significant cut down of the time complexity of the algorithm presented above (although it remains exponential since all configurations 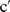 of all destination attraction basins 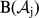 are still run through). This new algorithm has the advantage to decrease the transient time along the trajectories until the attractors, *i.e.* to accelerate their reaching without changing neither their nature nor their number nor their localisation. On this basis, we performed other simulations whose results lead to the same conclusion, that is: the robustness of the floral development process springs only in presence of gibberellin.

## Discussion

Concerning our toy network, the objective aiming at showing that the presence of gibberellin is necessary for the flower of the plant *Arabidopsis thaliana* to develop normally has been reached. By using attraction basins as gauges of the robustness of the system against influences of its periphery, we have highlighted, under the hypothesis of uniform random choice of initial configurations, the influence of gibberellin on the dynamical behaviour of the genetic regulatory network modeling the floral morphogenesis of this plant. The presence of this hormone harmonises the relative sizes of the floral attraction basins and allows the reduction of the relative distances between attraction basins. Moreover, it emphasises the significant increase of the robustness of the floral morphogenesis mechanisms and of the reproductive function of the plant. This approach, based on the idea that attraction basins are pertinent gauges for analysing in a theoretical framework robustness of a complex systems, can be generalised to draw a better understanding of the dynamical behaviour and the robustness of, for instance, formal neural networks and epidemiological systems an perhaps even many more discrete dynamical systems, provided they are not too big in practice. Indeed, as we have already mentioned, the time complexity of the algorithms underlying our method grows exponentially with the number of vertices in the interaction graphs of systems studied since they generally involve running through the exponential number of configurations of the system. This is of course a strong limitation (which cannot be bypassed unfortunately) since it disallows applying the method to large systems. Some techniques of data structures, such as the use of binary decision diagrams [48], [49] to encode configurations, could be of great interest to optimise our algorithms. Moreover, although it would mean loosing the exhaustivity property of the results, the implementation of the two different algorithms analysing robustness of a system against stochastic state perturbations as Monte-Carlo algorithms could certainly be of great interest to understand the impact of external or boundary constraints on the dynamical behaviour of large dynamical systems.

Let us come back to the biological framework of the floral morphogenesis of the plant *Arabidopsis thaliana* and open the discussion on the model of genetic regulation network proposed here.

First, let us note that the results we obtained depend of course entirely on the model of genetic regulation network from which they ensue. Thus, it is important to rule on the relevance of this model. We believe that it does propose a relevant approximation of the reality of the floral morphogenesis of the plant *Arabidopsis thaliana*. One argument in favour of this is precisely that from our analysis, we have derived that the model does indeed capture the importance of the presence of gibberellin. Nevertheless, this model remains an approximation that could certainly be improved.

In [35], the authors suggested to let the original network evolve with a particular block-sequential iteration mode characterised by the following ordered partition 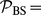
({emf1, tfl1},{lfy, ap1, cal}, {lug, ufo, bfu}, {ag, ap3, pi}, {sup}) by arguing that it is justified by the biological nature of the genetic regulation. However, as discussed earlier, with the current state of biological knowledge on this question, it is impossible to prove that this is indeed the iteration mode “selected” by nature. Besides, if the attraction basins are appropriate gauges to measure the robustness of a dynamical system, as we claim, comparing the relative sizes of attraction basins for different iteration modes gives an interesting argument in disfavour of this precise block-sequential iteration mode. Indeed, we find that when the sequential iteration mode defined by the following ordered partition 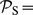
({emf1}, {tfl1}, {lfy}, {ap1}, {cal}, {lug}, {ufo}, {bfu}, {ag}, {ap3}, {pi}, {sup}) is used (as it has been done in this work), the proportions of the attraction basins corresponding to floral tissues are significantly greater than when the block-sequential iteration mode of [35] is used (see Table 7). In particular, let us note that the relative sizes of the carpel and the stamens attraction basins are three times larger with the sequential iteration mode. This agrees with a reinforced breeding tissues essential to the survival of the species.

**Table 7:** Iteration modes and relative sizes.

Attractor	Mendoza & Alvarez-Buylla block-sequential iteration mode 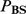	Sequential iteration mode chosen in the study 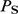
Sepal	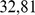 %	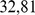 %
Petal	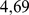 %	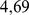 %
Carpel	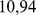 %	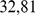 %
Stamen	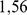 %	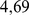 %
Inflorescence	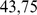 %	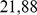 %
Mutant	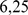 %	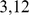 %

Relative sizes (in percents) of the attraction basins of the original Mendoza & Alvarez-Buylla network in the case where the block-sequential iteration mode 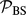 is used and in the case where the sequential iteration mode 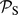 is used.

As we explained earlier, using a sequential iteration mode ensures that all attractors are fixed points. The specific sequential iteration mode 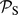 however, was chosen arbitrarily among the 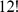 existing sequential iteration modes. And although we have theoretical arguments to explain why we think 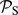 is more pertinent than 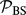, we do not claim that sequential iteration modes are the most pertinent iteration modes. There might indeed exist other iteration modes that also yield only fixed points and that are more realistic biologically. This leads us to the following question: amongst the 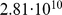 existing iteration modes of the original Mendoza & Alvarez-Buylla network, which are the ones that yield dynamics with only the six known fixed points while inducing non-floral attraction basins of minimal sizes and floral attraction basins whose sizes are ideally equilibrated? Assuming that over time, nature has “chosen” to maximise the probabilities for the plant *Arabidopsis thaliana* to develop as well as possible, we can consider that the iteration modes described in this question are amongst the realistic. Finding these iteration modes would solve the problem of choosing rightly the iteration mode.

Another way of avoiding this problem would be to show that precisely, iteration modes do not matter so much in the sense that the dynamical behaviour of networks are robust against changes of there iteration mode. Let us clarify this idea. We have argued that the limit cycles of the Mendoza & Alvarez-Buylla network observed with the parallel iteration mode are highly unlikely because if errors are allowed to be made in the updating order, then the network has little chance to land in a configuration corresponding to one of these cycles and, in addition, if ever it does, it has little chance to remain in the cycle. Thus, the limit cycles of the network observed with the parallel iteration mode display a kind of *instability*. On the contrary, some attractors such as fixed points are known to be *maximally stable* because there is no way of updating the states of all or of a part of the network elements to cause to leave the attractor. Generalising this argument to all iterations modes of a network, one may find that in some cases, the *stable* attractors of a network do not depend on the choice of the iteration mode.

From the more general point of view of the theory of biological regulation networks, we have shown the influence that boundary conditions may have on a regulation network modeled by a threshold Boolean automata network. However, other formalisms, such as those of Kauffman [30], [50] and Thomas [31], exist and we think that it would be of interest to dive our work on the robustness of regulation networks in the frameworks of Kauffman and Thomas. As they are defined, the frameworks of Kauffman and Thomas allow no easy way to modify voluntarily external or boundary variables. Nevertheless, one relevant idea is to represent these modifications by input data flows and to formally link these flows to output observation variables. This kind of study may call for a more general definition of the boundary and perhaps even of the environment of genetic regulation networks, such as those whose updating is ruled by the chromatin dynamics [51] or also neural networks with synchronising or desynchronising inputs [52]. Hopefully, it would also uncover new properties of theoretical and real biological networks.

In conclusion, let us evoke one last perspective that concerns explanation of biological phenomena and help in experimental choices. Traditionally, techniques coming from the theories of automata networks [36], [39], [53], [54], temporal logic and model checking [55]–[57], Petri nets [58], [59] and constraint logic programming [60], [61] have been applied to build an increased understanding of the behaviours of biological systems. As this article has endeavoured to show, these systems can also be formally studied by highlighting how a whole network can strongly depend on a singular element. Both approaches, however different, aim at deepening our understanding of some observed biological phenomena and we believe that both could be adapted into a useful tool for biologists helping them to choose what relevant experiments to perform.
